# Retraction: Efficacy of Zn-Aspartate in comparison with ZnSO_4_ and L-Aspartate in amelioration of drought stress in maize by modulating antioxidant defence; osmolyte accumulation and photosynthetic attributes

**DOI:** 10.1371/journal.pone.0281853

**Published:** 2023-02-10

**Authors:** The PLOS ONE Editors

This article [1] was identified as being linked to a series of submissions for which PLOS noted concerns about authorship and potential competing interests.

In addition, we noted the following concerns:

- The article’s figures are of poor resolution, and error bars appear similar across groups in several figures.
- Figs 3 and 4 appear to be duplicates and the legend for Fig 3 does not align with the figure contents.
- There are many instances in which p values are reported as 0.
- Primary data were not provided with the article, contrary to the Data Availability Statement.

The authors provided higher resolution figures, including a replacement for Fig 3. They clarified that p values less than 0.001 were reported as 0.

The authors also stated that the individual-level data are no longer available. Consequently, the article does not comply with the PLOS Data Availability Policy and the error bar concerns cannot be resolved.

The *PLOS ONE* Editors retract this article due to the unresolved concerns.

The authors did not reply to the retraction notification or could not be reached.
